# Supplementary material for: Seven inducible promoters for Zymomonas mobilis
Source: Microb Cell Fact. 2026 May 7;25:123. doi: 10.1186/s12934-026-03013-x (PMC13154678; doi:10.1186/s12934-026-03013-x)
Supplement: Supplementary file 1 — Supplementary Material 1. [file 12934_2026_3013_MOESM1_ESM.docx]

# Supporting information file 1 for

# Seven inducible promoters for *Zymomonas mobilis*

Gerrich Behrendt*

Analysis and Redesign of Biological Networks, Max Planck Institute for Dynamics of Complex Technical Systems, 39106 Magdeburg, Germany

*Corresponding author: gerrich.behrendt@gmail.com


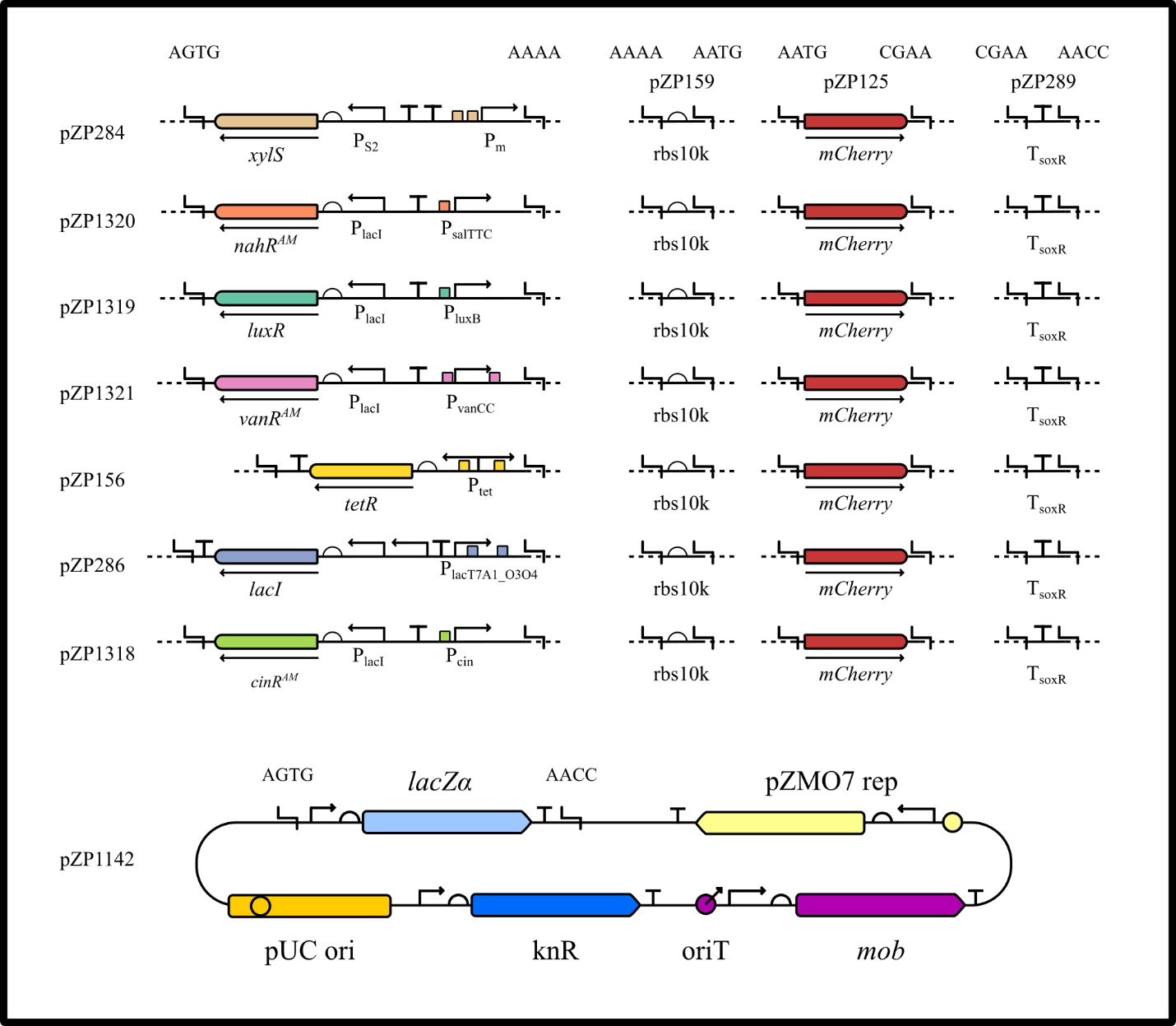


Supplemental Figure S1: Graphical cloning schematic of assembly parts used in this study. SBOL standardized glyphs are used.


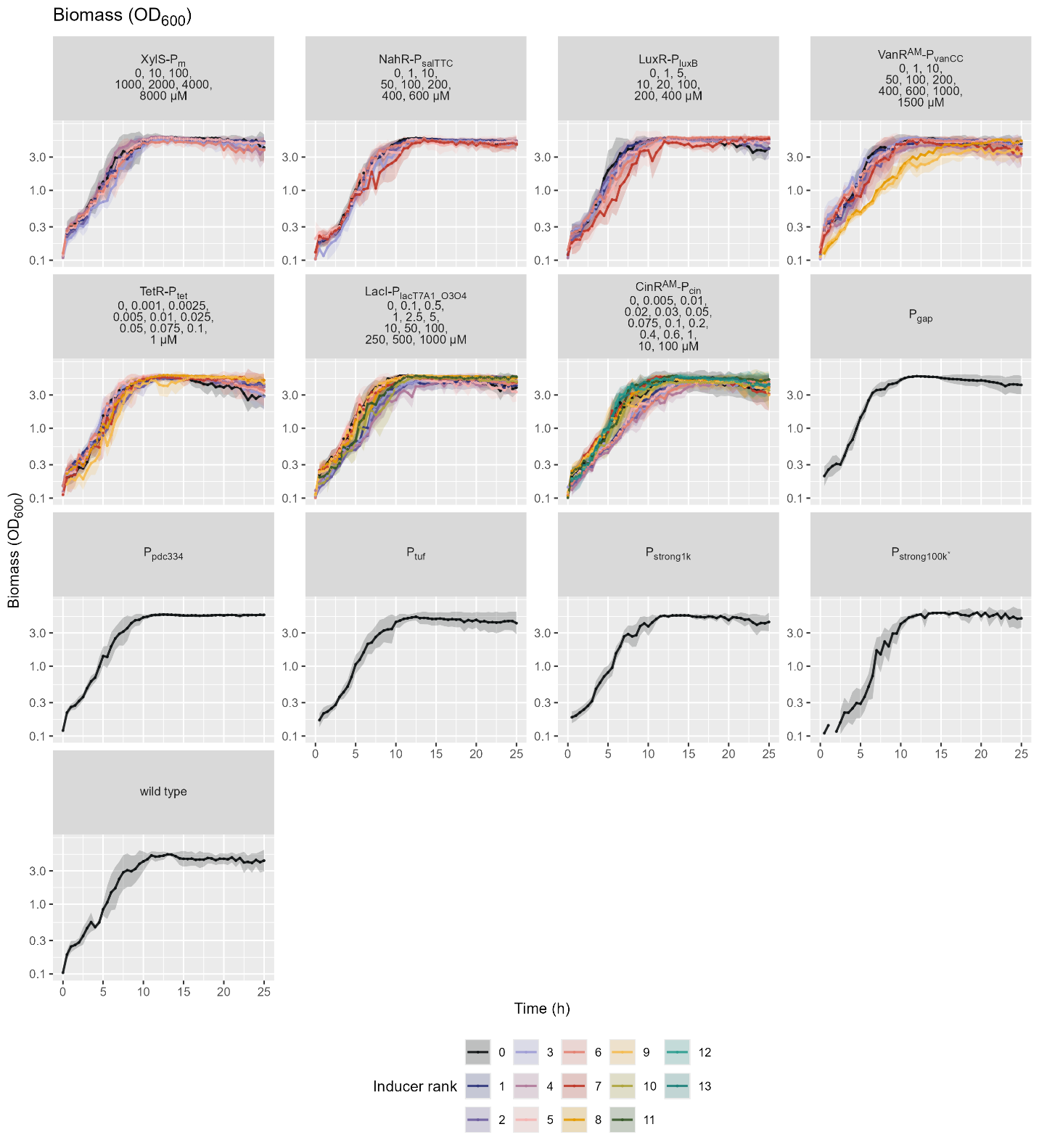


Supplemental Figure S2: Growth of all VANTAstar cultivations carried out in this study. Shown are two-sided 95% confidence intervals with the geometric mean. Inducers are only labeled by rank to obscure the different concentration ranges and instead indicate and increase or decrease of concentration for each compound. See Tables S1 and S2 for references to inducers and their concentrations.


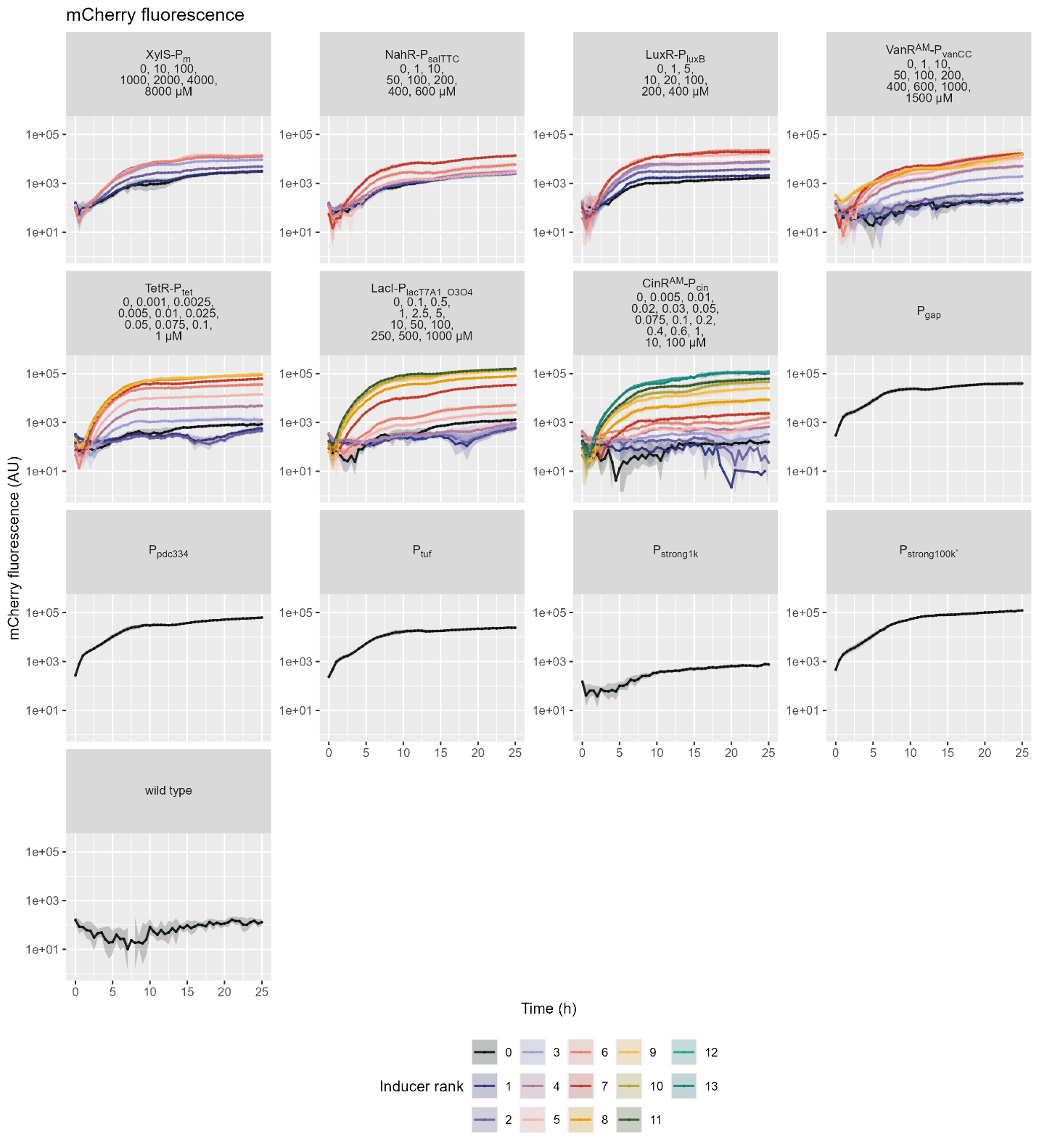


Supplemental Figure S3: mCherry fluorescence of all VANTAstar cultivations carried out in this study. Shown are two-sided 95% confidence intervals with the geometric mean. Inducers are only labeled by rank to obscure the different concentration ranges and instead indicate and increase or decrease of concentration for each compound. See Tables S1 and S2 for references to inducers and their concentrations.


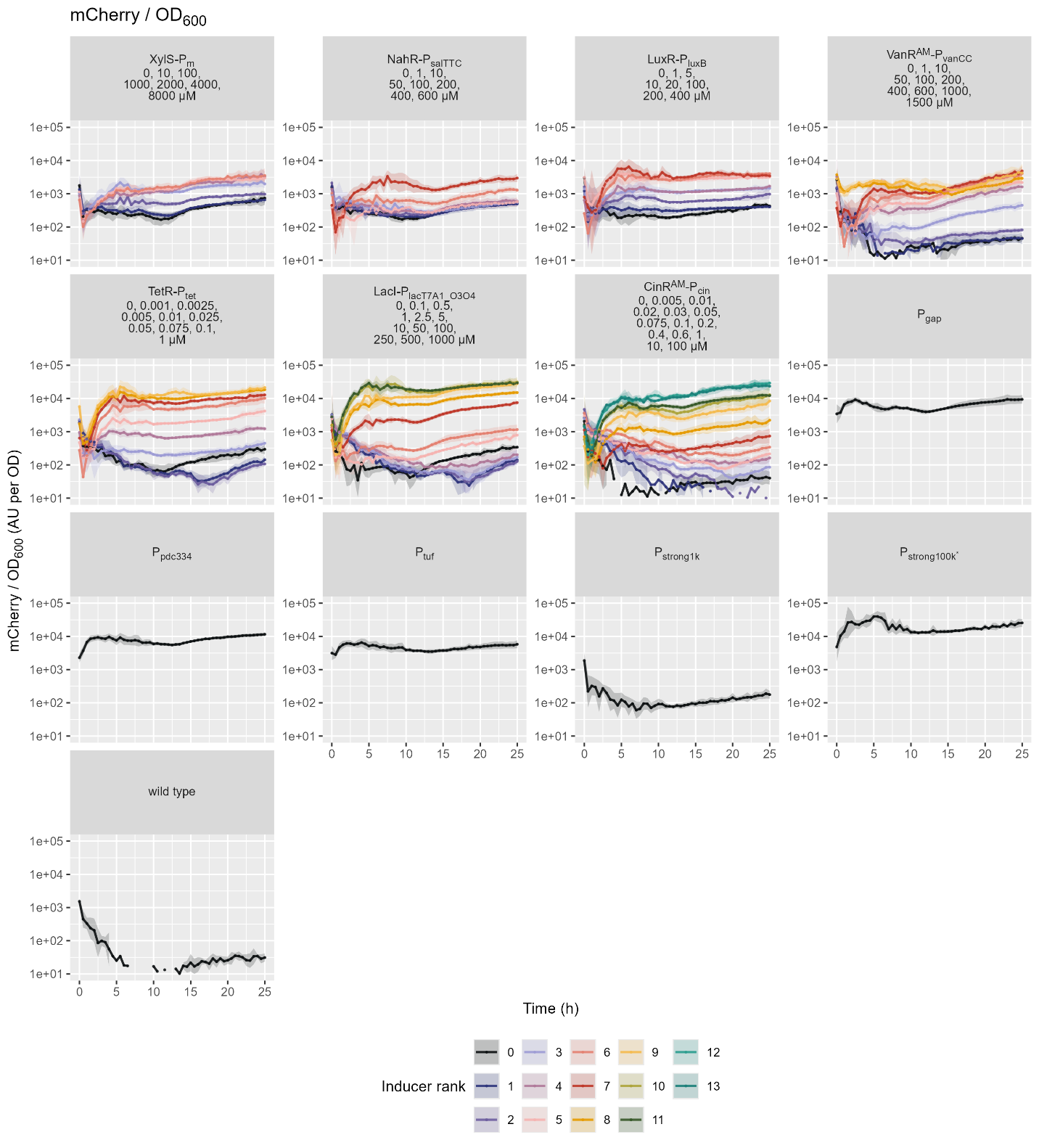


Supplemental Figure S4: mCherry fluorescence normalized to OD_600_ of all VANTAstar cultivations carried out in this study. Shown are two-sided 95% confidence intervals with the geometric mean. Inducers are only labeled by rank to obscure the different concentration ranges and instead indicate and increase or decrease of concentration for each compound. See Tables S1 and S2 for references to inducers and their concentrations.


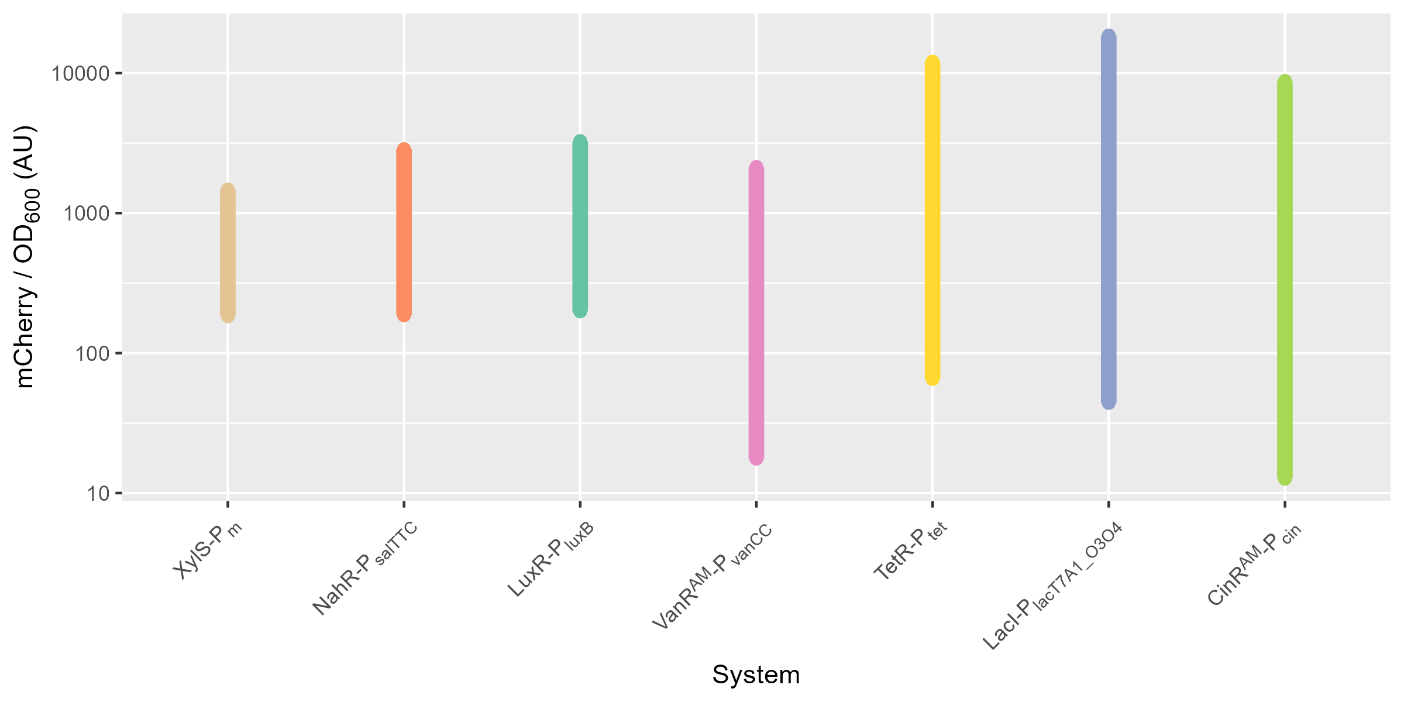


Supplemental Figure S5: Dynamic range of mCherry fluorescence over time for each inducible transcription system at the 10 h mark.


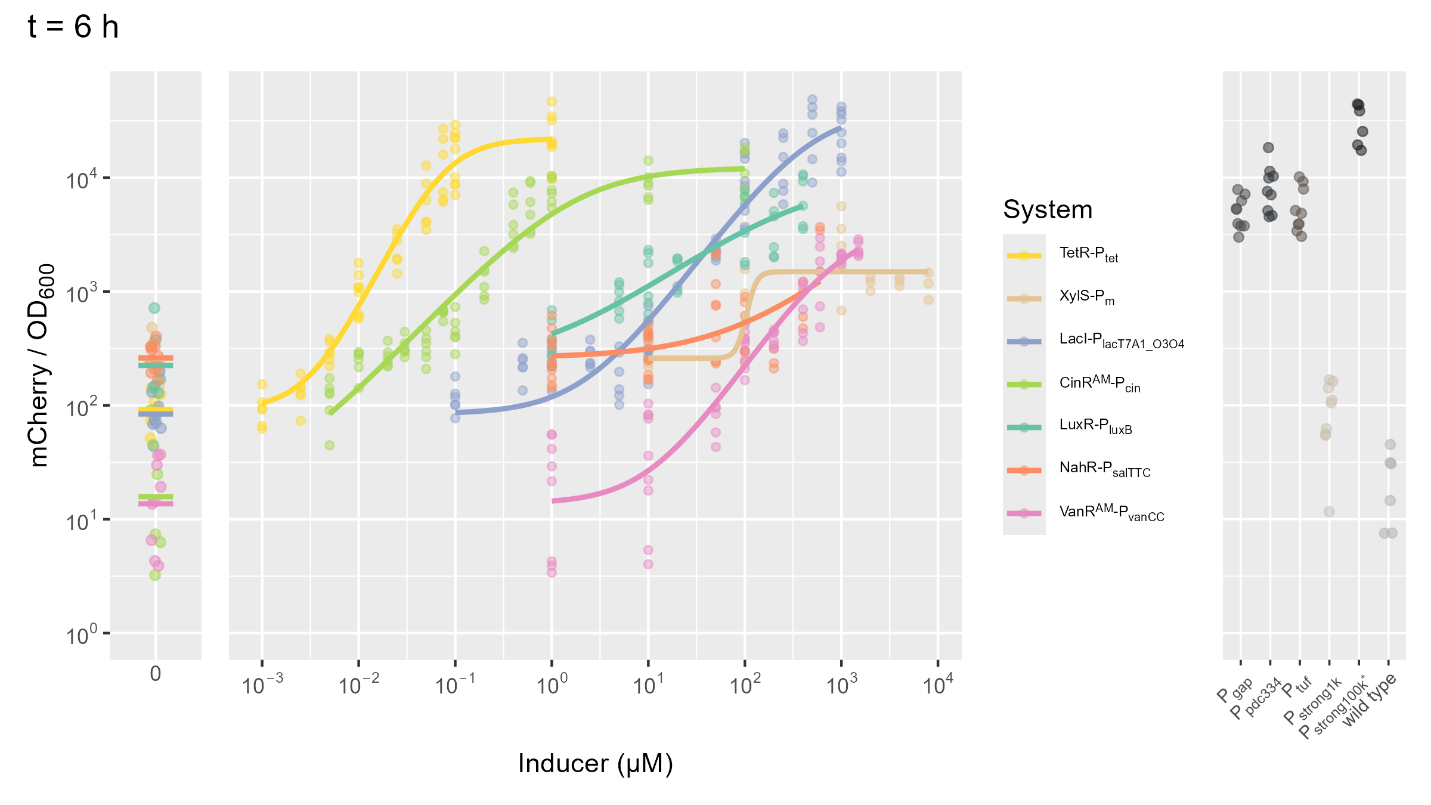


Figure S6: Titration curves with Hill fit and points of reference. The six-hour mark of the cultivation was used as a reference point to generate the plot. Each replicate is shown as a single dot.


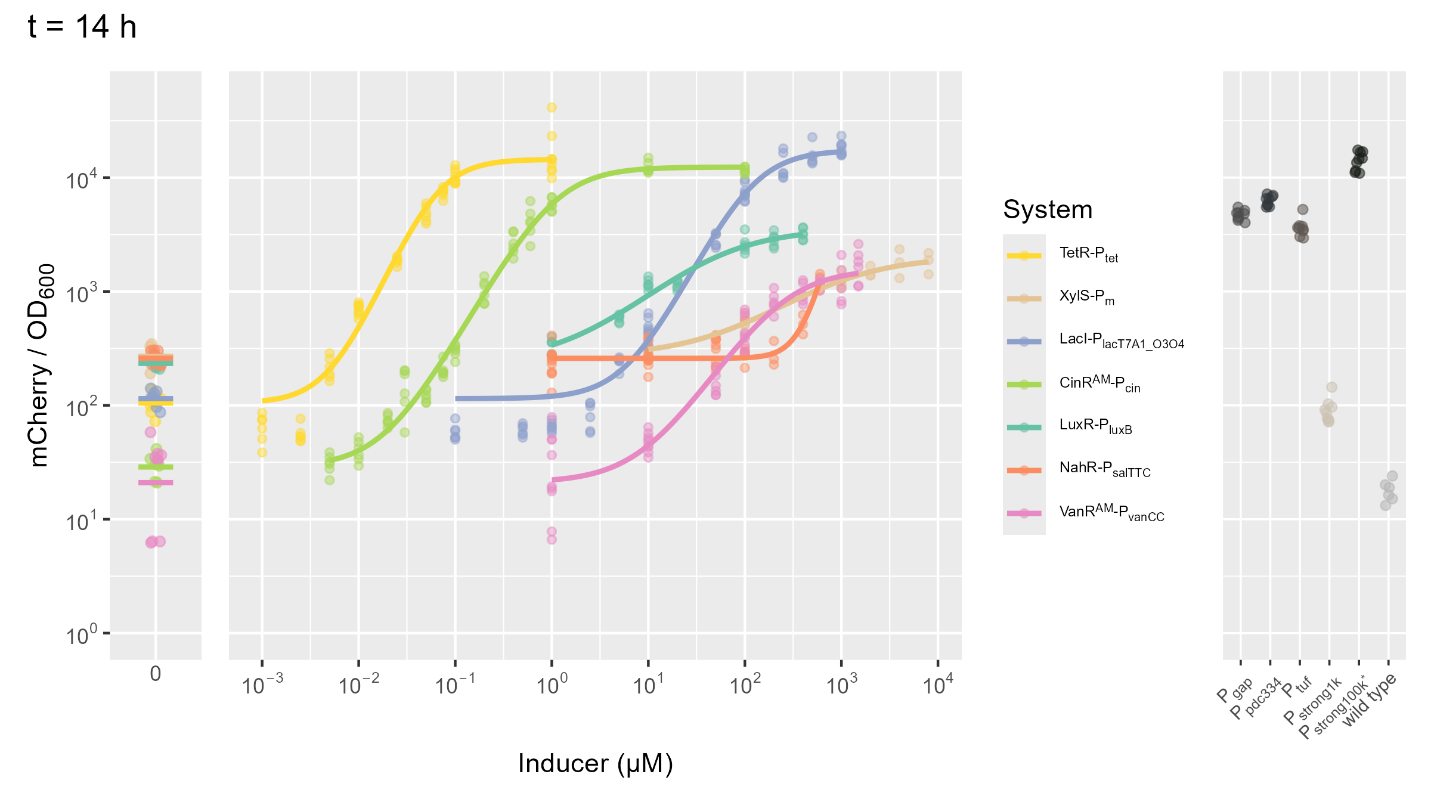


Figure S7: Titration curves with Hill fit and points of reference. The fourteen-hour mark of the cultivation was used as a reference point to generate the plot. Each replicate is shown as a single dot.


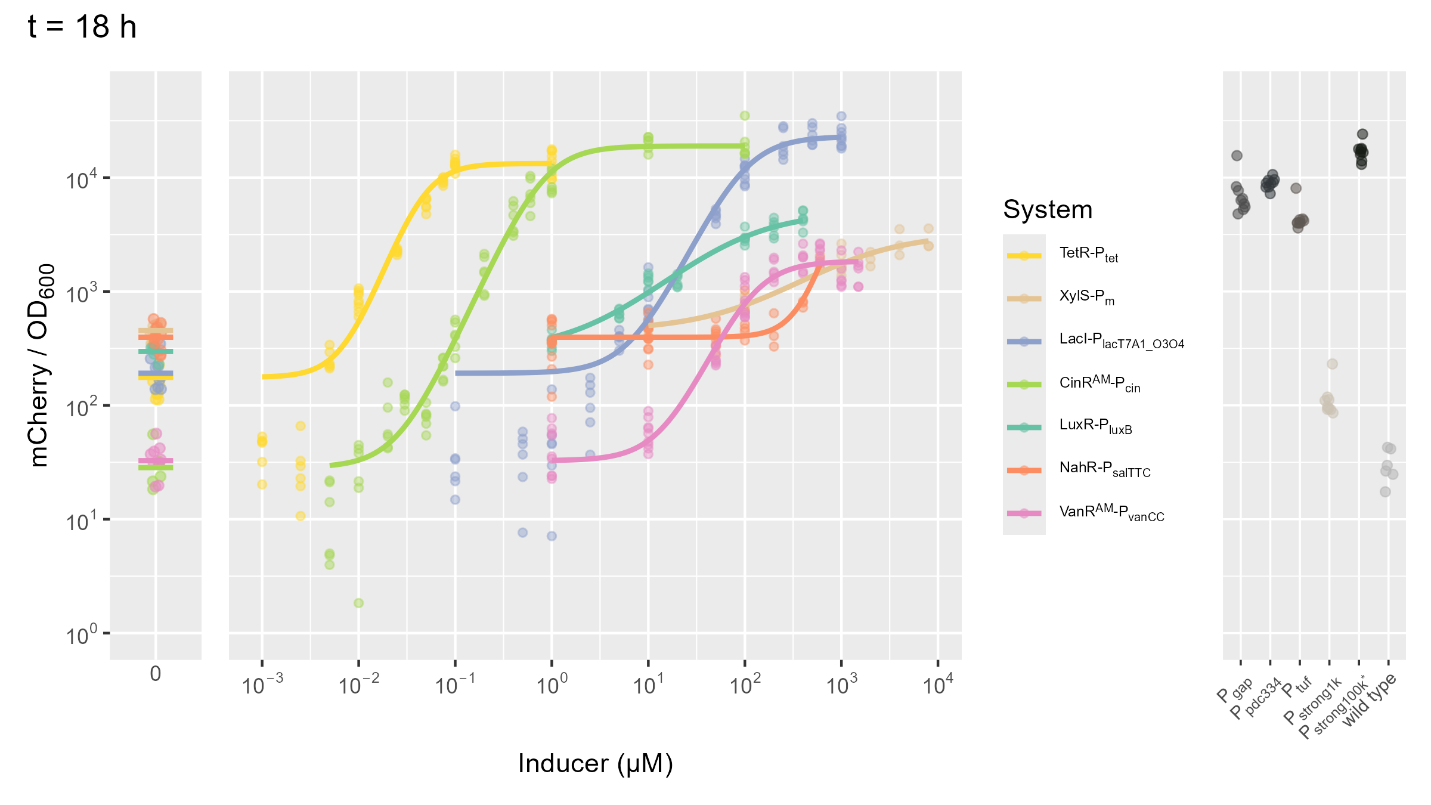


Figure S8: Titration curves with Hill fit and points of reference. The eighteen-hour mark of the cultivation was used as a reference point to generate the plot. Each replicate is shown as a single dot.


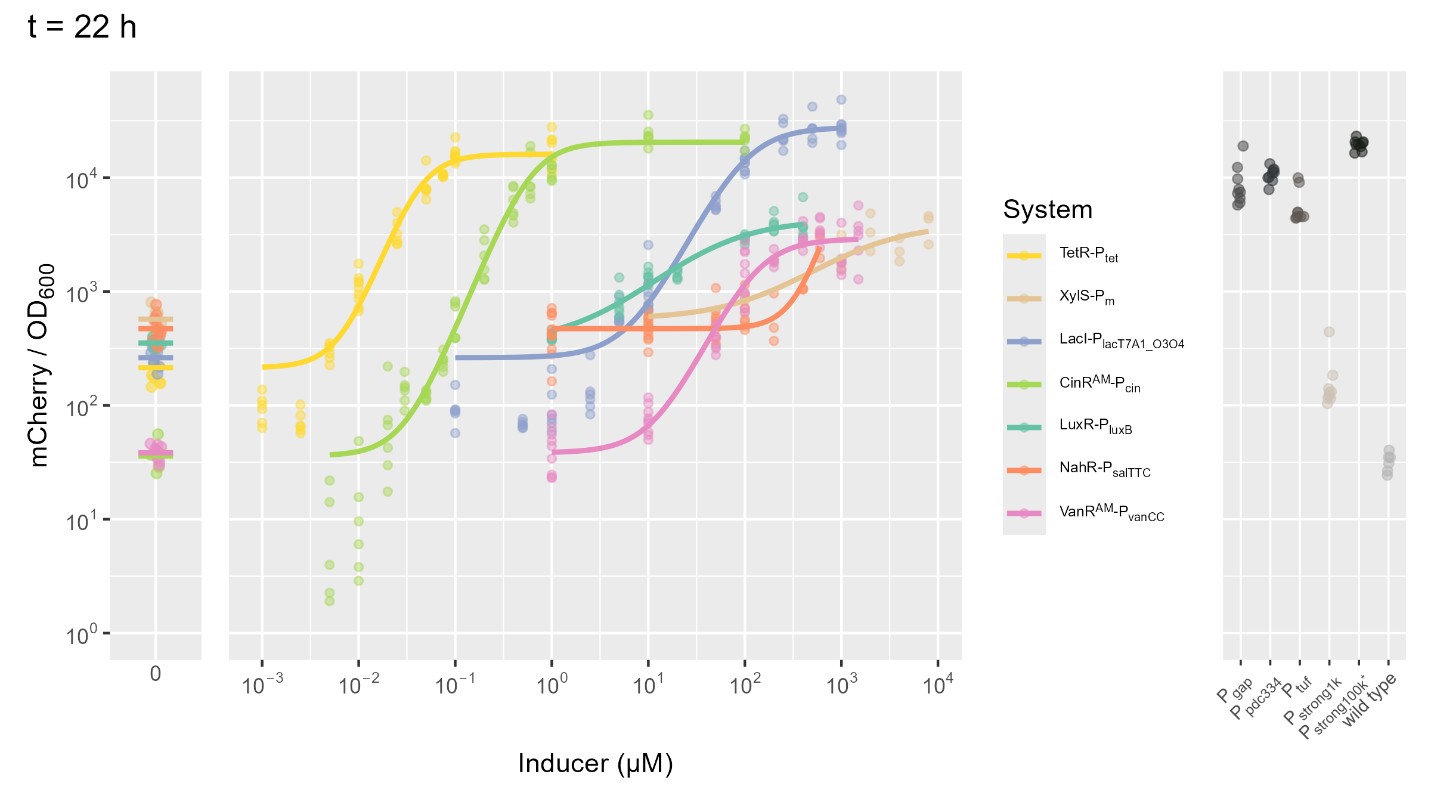


Figure S9: Titration curves with Hill fit and points of reference. The twenty two-hour mark of the cultivation was used as a reference point to generate the plot. Each replicate is shown as a single dot.

Supplemental Table S1: Substances used as inducers for the respective transcriptional regulators. Stocks were made either at 1000-times the applied inducer concentration (see supplemental Table S2) or the maximal solubility.

| **System** | **Inducer** | **Source** | **Solvent** |
| --- | --- | --- | --- |
| XylS-P_m_ | m-toluic acid (m-Tol) | Sigma, T36609 | Dimethyl sulfoxide (DMSO) |
| NahR-P_salTTC_ | Salicylic acid (Sal) | Sigma, 247588 | Water |
| LuxR-P_luxB_ | N-(3-oxohexanoyl) homoserine lactone (OC6) | Sigma, K3007 | Dimethyl sulfoxide (DMSO) |
| VanR^AM^-P_vanCC_ | Vanillic acid (Van) | Sigma, H36001 | Ethanol |
| TetR-P_tet_ | Anhydrotetracycline (aTc) | Sigma, 37919 | Water |
| LacI-P_lacT7A1_O3O4_ | Isopropyl-β-D-thiogalactopyranoside (IPTG) | Carl ROTH, CN08 | Water |
| CinR^AM^-P_cin_ | 3-Hydroxytetradecanoyl-homoserine lactone (OHC14) | Sigma, 51481 | Dimethyl sulfoxide (DMSO) |

Supplemental Table S2:Numbered inducer concentrations tested for each of the transcription regulators as µM. Meant to help the readability of Figures S2, S3 and S4.

| **Plasmid** | **Ind_0** | **Ind_1** | **Ind_2** | **Ind_3** | **Ind_4** | **Ind_5** | **Ind_6** | **Ind_7** | **Ind_8** | **Ind_9** | **Ind_10** | **Ind_11** | **Ind_12** | **Ind_13** |
| --- | --- | --- | --- | --- | --- | --- | --- | --- | --- | --- | --- | --- | --- | --- |
| XylS-P_m_ | 0 | 10 | 100 | 1000 | 2000 | 4000 | 8000 | 16000 | NA | NA | NA | NA | NA | NA |
| NahR-P_salTTC_ | 0 | 1 | 10 | 50 | 100 | 200 | 400 | 600 | NA | NA | NA | NA | NA | NA |
| LuxR-P_luxB_ | 0 | 1 | 5 | 10 | 20 | 100 | 200 | 400 | NA | NA | NA | NA | NA | NA |
| VanR^AM^-P_vanCC_ | 0 | 1 | 10 | 50 | 100 | 200 | 400 | 600 | 1000 | 1500 | NA | NA | NA | NA |
| TetR-P_tet_ | 0 | 0.001 | 0.003 | 0.005 | 0.01 | 0.025 | 0.05 | 0.075 | 0.1 | 1 | NA | NA | NA | NA |
| LacI-P_lacT7A1_O3O4_ | 0 | 5 | 10 | 50 | 100 | 250 | 500 | 1000 | 0.1 | 0.5 | 1 | 2.5 | NA | NA |
| CinR^AM^-P_cin_ | 0 | 0.005 | 0.01 | 0.02 | 0.03 | 0.05 | 0.075 | 0.1 | 0.2 | 0.4 | 0.6 | 1 | 10 | 100 |

Supplemental Table S3: Details of the titrations for all inducible transcription systems tested in this work, expanded with results from Meyer et al. 2019 for *E. coli* DH10B (blue) and Kostanjšek et al. 2026 for *R. sphaeroides* (red). The dynamic range for *E. coli* DH10B was calculated from the y_min_ y_max_ values for fluorescence provided by Meyer et al. 2019. The EC_50_ value is described as K in Meyer et al. 2019.

| **System** | **Hill slope** | **EC_50_ (/K) [µM]** | **Dynamic range [fold]** | **y_min_ / y_Pstrong1k_** | **y_max_ / y_Pstrong1k_** |
| --- | --- | --- | --- | --- | --- |
| XylS-P_m_ | 1.1 ± 0.31 | 238 ± 1.2e+02 | 8.06 ± 1.7 | 1.99 ± 0.49 | 16 ± 3 |
| NahR-P_salTTC_ | 0.75 ± 0.34 | 1.69e+06 ± 1e+09 | 15.4 ± 3.5 | 2.02 ± 0.38 | 31 ± 8 |
| NahR-P_salTTC_ *E. coli* DH10B | 1.8 | 43 | 595 |  |  |
| NahR-P_salTTC_ *R. sphaeroides* | 1.89 ± 0.22 | 8.8 ± 0.5 | 136 ± 5.6 |  |  |
| LuxR-P_luxB_ | 0.88 ± 0.12 | 37.8 ± 16 | 16.5 ± 1.3 | 2.15 ± 0.36 | 36 ± 6 |
| LuxR-P_luxB_ *E. coli* DH10B | 1.8 | 0.12 | 542 |  |  |
| VanR^AM^-P_vanCC_ | 1.23 ± 0.13 | 606 ± 2.8e+02 | 121 ± 41 | 0.19 ± 0.07 | 23 ± 4 |
| VanR^AM^-P_vanCC_ *E. coli* DH10B | 2.3 | 26 | 1250 |  |  |
| VanR^AM^-P_vanCC_ *R. sphaeroides* | 0.91 ± 0.1 | 310 ± 60 | 432 ± 192 |  |  |
| TetR-P_tet_ | 1.88 ± 0.1 | 0.057 ± 0.0074 | 185 ± 39 | 0.71 ± 0.18 | 131 ± 22 |
| LacI-P_lacT7A1_O3O4_ | 1.28 ± 0.048 | 284 ± 55 | 421 ± 1.3e+02 | 0.48 ± 0.16 | 201 ± 34 |
| LacI-P_lacT7A1_O3O4_ *R. sphaeroides* | 1.55 ± 0.22 | 59 ± 7 | 27 ± 2 |  |  |
| CinR^AM^-P_cin_ | 1.15 ± 0.053 | 1.29 ± 0.3 | 696 ± 3e+02 | 0.14 ± 0.06 | 96 ± 15 |
| CinR^AM^-P_cin_ *E. coli* DH10B | 2.3 | 0.43 | 500 |  |  |

Supplemental Table S4: Details of the titration results for each inducible transcription system, six hours into cultivation.

| **System** | **Hill coefficient** | **EC_50_ (/K) [µM]** | **Dynamic range (fold)** | **y_min_ / y_Pstrong1k_** | **y_max_ / y_Pstrong1k_** |
| --- | --- | --- | --- | --- | --- |
| XylS-P_m_ | 8.88 ± 3.7e+07 | 114 ± 6.4e+07 | 4.36 ± 1 | 3.35 ± 1.1 | 14.6 ± 4.7 |
| NahR-P_salTTC_ | 0.712 ± 0.6 | 2.49e+06 ± 2.7e+09 | 10.2 ± 3 | 3.37 ± 0.97 | 34.4 ± 14 |
| LuxR-P_luxB_ | 0.683 ± 0.17 | 287 ± 6.1e+02 | 29.1 ± 10 | 2.89 ± 1.1 | 84 ± 29 |
| VanR^AM^-P_vanCC_ | 1.23 ± 0.26 | 989 ± 1.2e+03 | 172 ± 51 | 0.176 ± 0.071 | 30.4 ± 8.6 |
| TetR-P_tet_ | 1.7 ± 0.11 | 0.077 ± 0.015 | 229 ± 46 | 1.18 ± 0.35 | 270 ± 89 |
| LacI-P_lacT7A1_O3O4_ | 1.14 ± 0.096 | 477 ± 2.8e+02 | 280 ± 61 | 1.08 ± 0.34 | 303 ± 98 |
| CinR^AM^-P_cin_ | 0.892 ± 0.053 | 1.67 ± 0.59 | 655 ± 3.7e+02 | 0.203 ± 0.12 | 133 ± 44 |

Supplemental Table S5: Details of the titration results for each inducible transcription system, 14 hours into cultivation.

| **System** | **Hill coefficient** | **EC_50_ (/K) [µM]** | **Dynamic range (fold)** | **y_min_ / y_Pstrong1k_** | **y_max_ / y_Pstrong1k_** |
| --- | --- | --- | --- | --- | --- |
| XylS-P_m_ | 0.859 ± 0.13 | 774 ± 3.9e+02 | 6.67 ± 1 | 3.04 ± 0.36 | 20.2 ± 3 |
| NahR-P_salTTC_ | 3.21 ± 2.7 | 2.84e+03 ± 2.3e+05 | 4.82 ± 0.52 | 2.92 ± 0.24 | 14.1 ± 1.8 |
| LuxR-P_luxB_ | 0.911 ± 0.083 | 43.4 ± 12 | 13.7 ± 0.89 | 2.63 ± 0.23 | 36.1 ± 3.2 |
| VanR^AM^-P_vanCC_ | 1.31 ± 0.13 | 244 ± 67 | 78.8 ± 26 | 0.236 ± 0.074 | 18.6 ± 2.9 |
| TetR-P_tet_ | 1.92 ± 0.14 | 0.0653 ± 0.011 | 148 ± 23 | 1.18 ± 0.12 | 175 ± 29 |
| LacI-P_lacT7A1_O3O4_ | 1.66 ± 0.11 | 124 ± 22 | 156 ± 11 | 1.29 ± 0.12 | 201 ± 17 |
| CinR^AM^-P_cin_ | 1.49 ± 0.045 | 1.08 ± 0.12 | 403 ± 45 | 0.324 ± 0.043 | 131 ± 10 |

Supplemental Table S6: Details of the titration results for each inducible transcription system, 18 hours into cultivation.

| **System** | **Hill coefficient** | **EC_50_ (/K) [µM]** | **Dynamic range (fold)** | **y_min_ / y_Pstrong1k_** | **y_max_ / y_Pstrong1k_** |
| --- | --- | --- | --- | --- | --- |
| XylS-P_m_ | 0.853 ± 0.14 | 1.16e+03 ± 6.7e+02 | 6.22 ± 0.83 | 4.15 ± 0.49 | 25.8 ± 4 |
| NahR-P_salTTC_ | 2.64 ± 2.3 | 4.03e+03 ± 4.2e+05 | 4.61 ± 0.45 | 3.62 ± 0.44 | 16.7 ± 2 |
| LuxR-P_luxB_ | 0.907 ± 0.09 | 70.4 ± 26 | 14.6 ± 1.3 | 2.72 ± 0.31 | 39.7 ± 4.8 |
| VanR^AM^-P_vanCC_ | 1.89 ± 0.16 | 120 ± 15 | 46.9 ± 7.6 | 0.299 ± 0.045 | 14 ± 2.1 |
| TetR-P_tet_ | 2.31 ± 0.44 | 0.0462 ± 0.014 | 76.2 ± 14 | 1.61 ± 0.31 | 123 ± 16 |
| LacI-P_lacT7A1_O3O4_ | 1.79 ± 0.32 | 97.5 ± 41 | 119 ± 15 | 1.75 ± 0.25 | 208 ± 25 |
| CinR^AM^-P_cin_ | 1.89 ± 0.12 | 0.817 ± 0.16 | 692 ± 1.4e+02 | 0.26 ± 0.049 | 180 ± 28 |

Supplemental Table S7: Details of the titration results for each inducible transcription system, 22 hours into cultivation.

| **System** | **Hill coefficient** | **EC_50_ (/K) [µM]** | **Dynamic range (fold)** | **y_min_ / y_Pstrong1k_** | **y_max_ / y_Pstrong1k_** |
| --- | --- | --- | --- | --- | --- |
| XylS-P_m_ | 0.938 ± 0.22 | 1.39e+03 ± 1.1e+03 | 6.53 ± 1.4 | 3.88 ± 0.7 | 25.3 ± 6 |
| NahR-P_salTTC_ | 2.58 ± 1.8 | 4.28e+03 ± 4e+05 | 5.64 ± 0.97 | 3.2 ± 0.53 | 18.1 ± 3.8 |
| LuxR-P_luxB_ | 0.975 ± 0.12 | 41.3 ± 14 | 10.9 ± 1.4 | 2.39 ± 0.36 | 26 ± 4.9 |
| VanR^AM^-P_vanCC_ | 1.8 ± 0.16 | 129 ± 20 | 77.1 ± 16 | 0.26 ± 0.041 | 20.1 ± 5.1 |
| TetR-P_tet_ | 2.31 ± 0.28 | 0.0427 ± 0.0085 | 78.9 ± 13 | 1.46 ± 0.28 | 115 ± 21 |
| LacI-P_lacT7A1_O3O4_ | 1.71 ± 0.22 | 99 ± 30 | 106 ± 11 | 1.78 ± 0.29 | 188 ± 32 |
| CinR^AM^-P_cin_ | 2.11 ± 0.23 | 0.61 ± 0.17 | 611 ± 77 | 0.244 ± 0.045 | 149 ± 24 |
